# Optimization of Gonyautoxin1/4-Binding G-Quadruplex Aptamers by Label-Free Surface-Enhanced Raman Spectroscopy

**DOI:** 10.3390/toxins14090622

**Published:** 2022-09-06

**Authors:** Yan Liu, Chengshun Jiang, Menghua Song, Yongbing Cao, Qiang Huang, Feng Lu

**Affiliations:** 1 Department of Pharmaceutical Analysis, School of Pharmacy, Naval Medical University, Shanghai 200433, China; 2 Shanghai Key Laboratory for Pharmaceutical Metabolite Research, Naval Medical University, Shanghai 200433, China; 3 Department of Pharmaceutical Analysis, School of Pharmacy, Fujian University of Traditional Chinese Medicine, Fuzhou 350108, China; 4 State Key Laboratory of Genetic Engineering, Shanghai Engineering Research Center of Industrial Microorganisms, MOE Engineering Research Center of Gene Technology, School of Life Sciences, Fudan University, Shanghai 200438, China; 5 Multiscale Research Institute of Complex Systems, Fudan University, Shanghai 201203, China; 6 Institute of Vascular Disease, Shanghai TCM-Integrated Hospital, Shanghai University of Traditional Chinese Medicine, Shanghai 200082, China

**Keywords:** surface-enhanced Raman spectroscopy, G-quadruplex aptamers, gonyautoxin, two-dimensional correlation spectroscopy, microscale thermophoresis, molecular dynamics

## Abstract

Nucleic acids with G-quadruplex (G4) structures play an important role in physiological function, analysis and detection, clinical diagnosis and treatment, and new drug research and development. Aptamers obtained using systematic evolution of ligands via exponential enrichment (SELEX) screening technology do not always have the best affinity or binding specificity to ligands. Therefore, the establishment of a structure-oriented experimental method is of great significance. To study the potential of surface-enhanced Raman spectroscopy (SERS) in aptamer optimization, marine biotoxin gonyautoxin (GTX)1/4 and its G4 aptamer obtained using SELEX were selected. The binding site and the induced fit of the aptamer to GTX1/4 were confirmed using SERS combined with two-dimensional correlation spectroscopy. The intensity of interaction between GTX1/4 and G4 was also quantified by measuring the relative intensity of SERS bands corresponding to intramolecular hydrogen bonds. Furthermore, the interaction between GTX1/4 and optimized aptamers was analyzed. The order of intensity change in the characteristic bands of G4 aptamers was consistent with the order of affinity calculated using microscale thermophoresis and molecular dynamics simulations. SERS provides a rapid, sensitive, and economical post-SELEX optimization of aptamers. It is also a reference for future research on other nucleic acid sequences containing G4 structures.

## 1. Introduction

Nucleic acid aptamers are single-stranded oligonucleotides (ssDNA or RNA) screened from a specific oligonucleotide library using systematic evolution of ligands via exponential enrichment (SELEX) technology [1,2]. These aptamers fold into a variety of stable spatial conformations, such as stem ring, hairpin, false knot, and G-quadruplex (G4), and bind to target molecules through electrostatic interactions, van der Waals forces, hydrogen bonds, and other intermolecular forces, often with high affinity and specificity [3,4]. G4 is a secondary nucleic acid structure formed by sequences rich in guanine [5,6,7,8,9]. Bioinformatic studies show that there are approximately 370,000 genes that can form G4s in the human body, especially in the telomere end and oncogene promoter regions [8,9,10]. Moreover, recent studies have shown that G4s play an important role in some key biological processes, a variety of human genetic diseases, and cancer development [8,9,11,12,13]. Thus, great interest has been attracted to target G4 at the promoter for the treatment of human diseases [10,11,14,15,16,17], and have become promising candidates as biosensors [18,19,20,21,22].

It has become a recent trend to target G4 at the promoter for the treatment of human cancers. For example, a novel small molecule targeting G4 of c-MYC promoter called quarfloxin and a G4 nucleolin aptamer AS1411 has entered clinical trials for the treatment of neuroendocrine/carcinoid tumors and metastatic renal cell carcinoma, respectively [11,14]. Other typical ligand drugs of the G4, such as TMPyP4, quindoline, and telomestatin, also show good anticancer activity [10,15,16,17]. In recent years, G4 functional nucleic acids have attracted great interest as a multifunctional molecular toolbox. For example, the G4/Thioflavin T complex has been widely used as a fluorescent probe to detect the activity of ions, microRNAs, DNA-related nucleases, and aptamers, as well as that of histone acetylation-related enzymes, providing a sensing platform for high-throughput screening and evaluation of targeted inhibitors [18,19,20]. G4 also has the potential to construct fluorescent protein mimics [21,22]. Therefore, owing to the advantages of a wide targeting range, high affinity, and good specificity, aptamers have broad application prospects in basic biological research, analysis and detection, clinical diagnosis and treatment, and new drug research and development. In this study, we focus on aptamers containing a G4, so all the “aptamers” mentioned refer to G4 aptamers.

However, aptamers obtained using SELEX screening technology do not always have the best affinity or binding specificity to ligands. In recent years, primer-free SELEX, toggle SELEX, genomic SELEX, MB-SELEX, and GO-SELEX have been developed to improve the efficiency and specificity of SELEX screening [23,24,25,26,27,28]. At the same time, post-SELEX aptamer optimization strategies are constantly emerging, such as truncation, mutation, and cyclization, to obtain high-performance aptamers [4,26,29,30,31,32,33,34,35,36]. However, the theoretical and experimental basis of these optimization strategies is not mature enough, especially the induced-fit structure when the aptamer binds to the target is not fully considered.

In addition to the traditional physical and chemical methods, isothermal titration calorimetry, surface plasmon resonance, microscale thermophoresis (MST), and biolayer interferometry have been recently developed to investigate the interaction between aptamers and small molecule ligands, as well as to measure the main kinetic or thermodynamic parameters [37,38,39,40,41]. However, the limitations of these methods, such as labeling and fixing ligand molecules or aptamers, hardly provide the base sequence or structural information about the DNA chain. Detailed information on conformational changes remains unresolved, which precludes the understanding of ligand molecules and aptamer recognition mechanisms and subsequent optimization based on structure or conformation.

Surface-enhanced Raman spectroscopy (SERS) is a powerful analytical tool for molecular structure characterization and quantitative determination, applied to detect single-base mutations and sequence changes in single-stranded nucleic acid sequences, as well as study DNA conformation [42,43,44,45,46,47,48,49,50,51,52,53,54]. Researchers are interested in the aptamer-based SERS method, which mainly fixes the modified aptamer on the surface of nanoparticles or matrix, constructs a SERS biosensor to study the interaction between small molecules and aptamers, as well as achieves accurate quantitative analysis. Recently, Xu et al. proposed a label-free SERS method, introducing MgSO_4_ to promote the aggregation of polymerized iodide-modified Ag nanoparticles (Ag IMNPs), for detecting single-strand and double-helix DNA [49]. In addition, our team has applied this approach to study the conformational changes of a non-G4 aptamer binding to theophylline molecular during the binding process, which revealed that theophylline interacts with the aptamer and identified the bases through which this interaction occurs [40]. It is of great significance to determine the key binding groups and conformational changes during the binding process for guiding SELEX screening and aptamer optimization. Furthermore, the label-free SERS method developed in this paper can be used for the optimization of the G4 aptamer.

Gonyautoxin (GTX) 1/4 is a representative neurotoxin in paralytic shellfish poisoning, which is in the top three global marine biological hazards; it has the characteristics of super toxicity, quick effect, difficult detection, difficult prevention, and no effective treatment [55,56]. The aptamer of GTX1/4 screened by SELEX specifically recognized GTX1/4 with binding affinity (K_d_) in the range of nanomoles [26], which is worth further improvement, from tens of nanomoles to a few nanomoles or even a picomole. Furthermore, the aptamer has been confirmed as a G4 aptamer. The aptamers not only have the potential as recognition elements for the detection of marine biological toxins, but also may be further developed as candidate drugs for toxin antagonists. Therefore, it is particularly critical to guide the optimization of G4 aptamers for rapid detection and treatment of marine biotoxin poisoning.

In this study, we investigated the optimization of G4 aptamers of GTX1/4 using label-free SERS. The binding process of aptamers and GTX1/4 induced by the structure was discovered using SERS combined with two-dimensional correlation spectroscopy (2DCOS), which provided a structural basis for mutation and truncation optimization of G4 aptamers. Furthermore, the interaction strength between GTX1/4 and G4 was quantified by measuring the relative strength of SERS bands to quickly obtain an optimized aptamer with more affinity. Consequently, this label-free and non-fixed SERS method provides a sensitive, simple, fast, and economic analysis platform, which can not only guide SELEX optimization of G4 aptamers, but also propose optimization strategies such as mutation and truncation. Given the range of promising application of G4 aptamers, the method has a significant potential as a tool to aid design of G4 aptamers.

## 2. Results and Discussion

### 2.1. Analysis of the SERS Spectrum of Wild-Type Aptamer GO18

The basic principle of SERS detection of G-quadruplex DNA based on Mg^2+^ to polymerize iodide-modified Ag nanoparticles (Ag IMNPs) is that iodide ions act as a cleaning agent and makes the surface of Ag IMNPs negatively charged. The addition of magnesium ions not only neutralized the negative charge of the DNA phosphate skeleton but also allowed better aggregation of Ag IMNPs through electrostatic interactions [49]. 

**Figure 1:**
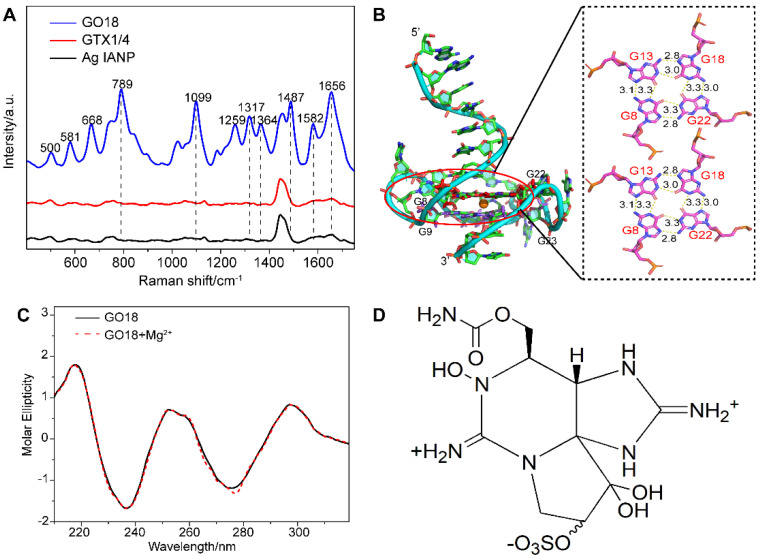
(**A**) SERS spectra of GO18, GTX1/4, and Ag IANP. Dashed lines indicate bands discussed extensively in the text. (**B**) Full-length three-dimensional model of wild-type aptamer GO18, and the hydrogen bond and bond length between G-tetrads of GO18 are given in the panel on the right. The specific number on the bond indicates the bond length, and the unit is Å. (**C**) CD spectra of GO18 without (solid line) and with magnesium ion (dash line). (**D**) Chemical structure of GTX1/4.

As a result, direct SERS signals of DNA were achieved under this method. Figure 1A shows the Raman spectra of aptamer GO18 (see Table S1 for the detailed sequences) using Ag IMNPs as SERS substrates, indicating that the established SERS method could detect the ssDNA signal well. According to the surface selection rules of SERS, when the vibrational atoms are close to the enhanced substrate surface, their Raman signals are enhanced. Two peaks at 789 and 1099 cm^−1^ were assigned to the stretching vibration of the PO_2_^−^ framework, indicating that the phosphate skeleton was close to the surface of the Ag IMNPs. It has been reported that the positions and intensities of the peaks in the range of 1300–1380 cm^−1^ are related to the types of glycolic bond angle (GBA) of guanine (G), adenine (A), and thymine (T) [57]. Therefore, the featured bands in this region could be used to distinguish the *anti* and *syn* GBA conformations of G4s. The peak at 1317 cm^−1^ could be assigned to dG C2’-endo/*anti* and the peak at 1356 cm^−1^ to dG C2’-endo/*syn*; *anti*-GBA formed a parallel G4 structure. We observed that the intensity of the peak at 1317 cm^−1^ was higher than that at 1364 cm^−1^, indicating that GBAs in the G4 structure of the aptamer tended to *syn* conformation, and the wild-type aptamer GO18 tended to a parallel structure.

From their SERS spectra, the featured bands corresponding to the formation of G4s were observed. Among them, the peaks at 1487, 1581, and 1656 cm^−1^ were attributed to the stretching vibration (ν) of the dG-N7 Hoogsteen H-bond, deformation vibration (δ) N2H interbase H-bond, and ν of O6 interbase H-bond, respectively, which were used to evaluate the stability of G4s [47,53]. The above results showed that the Ag IMNPs enabled the G4 structure of DNA to be placed in an appropriate “hot spot” position; therefore, a high-quality signal of the G4 was obtained. In addition, our method truly reflected the fingerprint information of the basic structure of DNA, including the base, phosphate skeleton, and the deoxyribose. Each band of the wild-type aptamer GO18 spectra was assigned referring to the literature [47,50,51,53,58,59,60,61] and is listed in Table S2 (see Supplementary Materials).

Molecular dynamics (MD) simulation has become an effective analytical method in structural biology, which can be used to study the interaction of molecules and their ligands, as well as the components of the molecule, and has become an indispensable supplement to many experimental methods [39,41,62,63,64]. The results of the MD simulation (Figure 1B) showed that the wild-type aptamer GO18 had a parallel structure, owing to the link between the Hoogsteen hydrogen bonds of two G-tetrad consisting of dG8, dG13, dG18, and dG22, as well as dG9, dG14, dG19, and dG23, resulting in high stability.

As shown in Figure 1C, GO18 had a strong positive peak at 260 nm and a negative peak at 240 nm, in agreement with the common knowledge that G4s are produced and stabilized by the presence of specific concentrations of monovalent cations. In addition, we studied whether the addition of 10 mM magnesium ions to Ag IMNPs would affect the formation and change the structures of the G4s. The CD spectra of G4s with or without the same concentration of magnesium ions presented in Ag IMNPs were obtained, showing no significant difference between the two measurement results, and no significant effect on either the peak position or the intensity of the characteristic bands. Therefore, the addition of magnesium ions did not change the natural structure of the DNA G4.

### 2.2. Interaction between Wild-Type Aptamer GO18 and GTX1/4

#### 2.2.1. SERS

Under the above conditions, the characteristic SERS signal of GTX1/4 was not detected. SERS still suffers from challenges in the detection of low-affinity molecules, which means that a high-affinity target to the surface of the NPs must be chosen to achieve sensitive detection [65]. As shown in Figure 1D, GTX1/4 is a carbamate alkaloid derivative from different substituents with the basic skeleton of saxitoxin. Ag NPs have a weak affinity for such molecules, which makes it difficult for GTX1/4 to bind to the surface of NPs and enter their “hot spots” [66]. Although GTX1/4 can bind to wild-type aptamer GO18 with high affinity, GO18 was free rather than fixed on the surface of NPs or functionalized with SERS substrates, which was still not enough to decrease the distance between GTX1/4 and NPs or obtain the SERS signal of GTX1/4 directly and sensitively.

**Figure 2:**
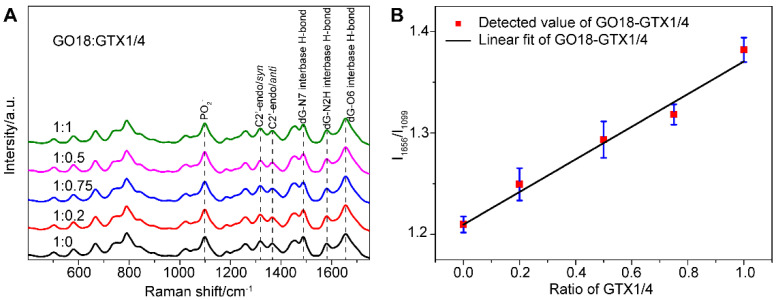
(**A**) SERS spectra of GO18 with different GTX1/4 ratios. The peaks of the PO_2_^−^ and featured bands corresponding to G4 of GO18 have been marked. All spectra were normalized to the PO_2_^−^ peak at 1099 cm^−1^. (**B**) The relative intensity ratio (I_1656_/I_1099_) was used to plot the GTX1/4 addition ratio. The error bar shows the standard deviation.

Figure 2A shows that the spectra of aptamers change before and after the addition of GTX1/4. This may be because aptamers can transform freely and flexibly to form the optimal spatial conformation and then bind with GTX1/4. Moreover, GTX1/4 has a relatively small molecular volume, resulting in a slight change in the conformation of aptamers. In the follow-up study, to see the difference, the PO_2_^−^ signal was used as the internal standard, and the spectra were normalized to the intensity of PO_2_^−^ to show the influence of GTX1/4 on the SERS signal of GO18.

From their SERS spectra, we observed that some changes took place in the featured bands corresponding to the formation of the G4. The SERS band at 1487 cm^−1^ was observed, which indicated that a hydrogen bond was formed between dG(N7) and (NH) in the G4, rather than between dG (N7) and water. In addition, the peak intensity of νdG (C6 = O6) at 1656 cm^−1^ increased gradually as well, which further confirmed that the binding site of GTX1/4 and GO18 was indeed on the G4 plane. At the same time, the relative intensity ratio (I_1656_/I_1099_) of G4-related SERS bands in the GTX1/4-aptamer complex was selected to plot the amount of GTX1/4, and a linear relationship was observed, as shown in Figure 2B, suggesting that the method we developed could be used to quantitatively detect the binding of ligand and aptamer.

#### 2.2.2. DCOS

**Figure 3:**
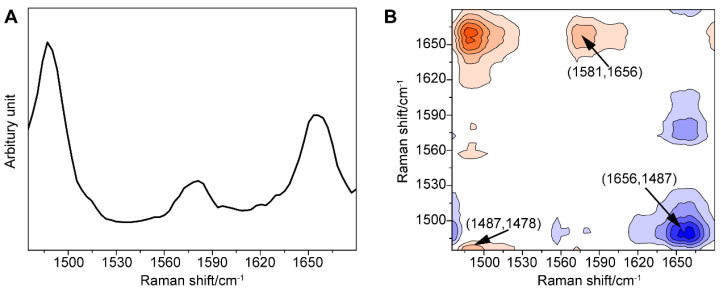
(**A**) Power spectrum of the GO18-GTX1/4 complex in the range of 1475–1680 cm^−1^ with the perturbation of GTX1/4 concentration. (**B**) Asynchronism spectrum of the GO18-GTX1/4 complex in the range of 1475–1680 cm^−1^ with the perturbation of GTX1/4 concentration. Contours shaded red indicate positive asynchronous peaks; contours shaded blue indicate negative asynchronous peaks.

Furthermore, we amplified the minor change in the one-dimensional (1D) SERS signal using the generalized 2DCOS method, thus providing the spectral fingerprint of the target molecule, which better explained the spectral variations induced by external perturbation, in this case, the concentration of GTX1/4. To our surprise, significant differences between the behaviors of the peaks were found (Figure 3).

First, the autopower spectrum extracted from the slice trace of the diagonal in the synchronous map represented the degree of spectral intensity changes and the susceptibility to external perturbation of the corresponding spectral variables. The autopower spectrum of the GO18-GTX1/4 complex (Figure 3A) developed strong autocorrelation peaks at 1487, 1581, and 1656 cm^−1^. Among them, the peak at 1487 cm^−1^ was the most sensitive to perturbation, although it was weak in the 1D SERS spectrum, followed by the peaks at 1656 and 1581 cm^−1^. These three positive autocorrelation peaks also illustrated that the intensity of the peak increased with the increase in the GTX1/4 ratio. As a result, we found that an increase in the number of hydrogen bonds was associated with the increased stability of the GO18-GTX1/4 complex. These three positive autocorrelation peaks showed that the intensity of the peak increased with the increasing concentration, and the number of hydrogen bonds increased accordingly, indicating that the stability of the GO18-GTX1/4 complex also increased.

Second, all the signs of cross-peaks generated in the synchronous maps were positive. According to the Noda rule [67], the signature of a cross-peak in the asynchronous map directly explains the sequential relationship between two relevant spectral variables. The asynchronous spectrum was antisymmetric with respect to the diagonal, and the transverse and longitudinal coordinates of the cross-peaks close to the diagonal distribution were similar, which indicated that the peak shift occurred. As shown in Figure 3B, the peak at 1487 cm^−1^ and the peaks within 1485 cm^−1^ and 1478 cm^−1^ all have positive cross-peaks, which indicates that the band at 1487 cm^−1^ has a red shift as the concentration increased. In other words, the peak shift that is not recognized in the power spectrum can be identified in the asynchronous spectrum. In addition, it should be noted that the cross-peaks away from the diagonal line represented the two spectral variables derived from the molecule with different vibration modes; for instance, the positive peaks at 1487 and 1581 cm^−1^, 1487 and 1656 cm^−1^, and 1581 and 1656 cm^−1^, as well as corresponding negative peaks at 1581 and 1487 cm^−1^, 1656 and 1487 cm^−1^, and 1656 and 1581 cm^−1^, indicated that the peaks at 1656, 1487, and 1581 cm^−1^ changed with the increasing concentration of GTX1/4. At the same time, we further revealed that the order of peak changes was 1487, 1581, and 1656 cm^−1^, and confirmed that the addition of GTX1/4 changed the conformation of the G4 aptamer, which led to a change in the SERS spectra.

As mentioned above, through autopower analysis, synchronous spectrum, and asynchronous maps, the weak changes, such as the intensity, displacement, and change order of the spectral peaks, were visually interpreted, and the change between characteristic peaks was better identified. Therefore, this combined strategy between SERS and 2DCOS was helpful in the study of the binding between GO18 and GTX1/4.

#### 2.2.3. MD Simulations

**Figure 4:**
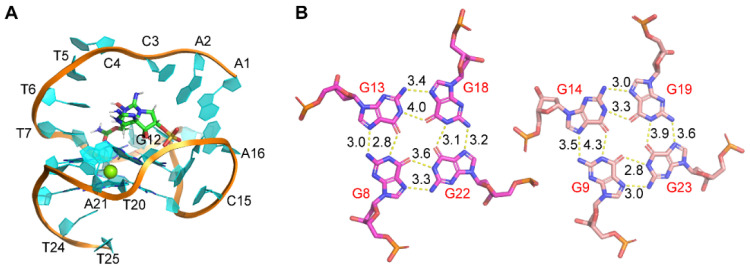
(**A**) The complex structure of GO18-GTX1/4 obtained by MD simulation. (**B**) Hydrogen bond and bond length between G-tetrads of the GO18-GTX1/4 complex. The specific number on the bond indicates bond length, and the unit is Å.

Here, we further studied the binding of GTX1/4 to GO18 using MD simulations (Figure 4). The results showed although the epimeric mixture GTX1/4 has different sulfonic acid group orientations, the sulfonic acid group orientations finally tend to be consistent under the induction of aptamers. GTX1/4 could spontaneously associate with GO18 to form stable conformations under the driving effect of van der Waals forces and hydrogen bonds, and the binding pocket resembled the combination of a platform and an arch-like structure. The platform was a G-tetrad consisting of G8, G13, G18, and G22, and the arch-like structure was at the 5’-end of GO18, which was stabilized by the π–π stacking between A1 and A6 and T7 and G22, respectively (Figure 4A). The average binding free energy of GO18-GTX1/4 complex was −10.07 kcal/mol, which led to the decrease in molecular energy so that the structure of the complex was more stable than that of GO18. By comparing the length of hydrogen bonds between G-tetrads and between GO18 (Figure 1B) and GO18-GTX1/4 complex (Figure 4B), we found that the hydrogen bond length between G13 (N2) and G18 (N7) and that between G22 (N2) and G8 (N7) became longer and the bond energy became smaller. This was also consistent with the results of cross-peaks in the asynchronous spectrum and red-shift of the characteristic peaks. At the same time, MD technology provided detailed dynamic information of aptamer binding to GTX1/4 at the atomic level.

The verification of 2DCOS and SERS, as well as that of SERS and MD simulations, showed that the SERS method could be used to characterize the structure of aptamers, obtain new insights into the interaction mechanism between aptamers and GTX1/4, and quantitatively analyze the interaction between aptamers and small molecules of the G4. Given the molecular-level analysis of the structure or conformation of substances and high sensitivity to the intermolecular interaction forces, SERS technology could characterize the interaction between aptamers and small molecules and reflect the information of key binding sites to guide further aptamer optimization.

### 2.3. Guide Mutation and Truncation Optimization of Aptamers

To further verify the universality of the method and obtain aptamers with different affinities to GTX1/4, we optimized the wild-type post-SELEX aptamers. Two common optimization methods were used as follows: Mutating the aptamer base; truncating the aptamer to further study the binding of the aptamer and GTX1/4.

#### 2.3.1. Optimization of Mutation 

Since the binding site of GTX1/4 with the aptamer is in the G4 plane, G12 and T7 belong to its junction position, which may be of great significance for maintaining the conformation and stability of the G4 plane. For the base mutation of aptamers, four mutated aptamers were obtained by mutating 7’- and 12’-bases (see Table S1 for the detailed sequences for the related mutated aptamers). Except for the different aptamer sequences, all other detection conditions were the same, to ensure comparability of the results.

**Figure 5:**
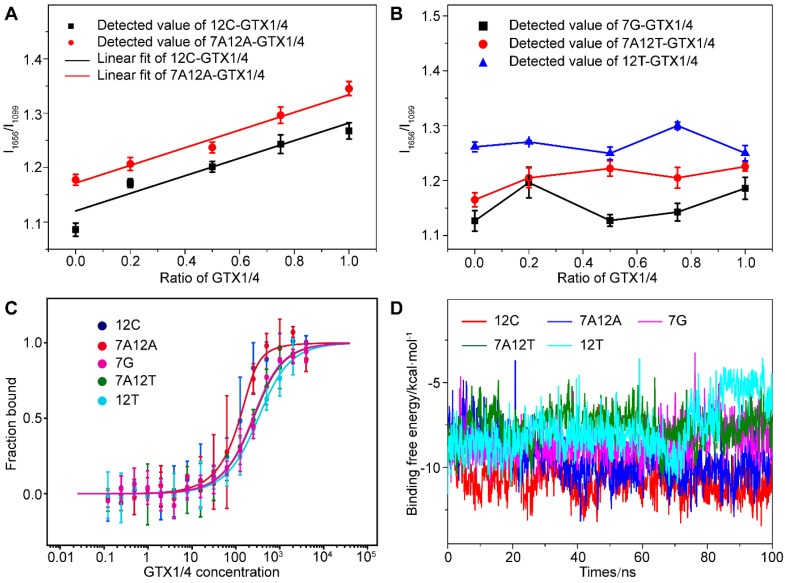
(**A**) The graph of relative intensity ratio (I_1656_/I_1099_) of two mutant aptamers (12C and 7A12A) binding to GTX1/4 versus the addition ratio of GTX1/4. The error bar shows the standard deviation. (**B**) The relative intensity ratio (I_1656_/I_1099_) of three mutant aptamers (7G, 7A12T, and 12T) with different GTX1/4 ratios. (**C**) MST experiments of five mutant aptamers (12G, 7A12A, 7G, 7A12T, and 12T). (**D**) Time-dependent binding free energy of mutant aptamers (12G, 7A12A, 7G, 7A12T, and 12T) binding to GTX1/4 by MD simulation.

We found that when dG12 was mutated to C (12C), dT7 and dG12 changed to A (7A12A), there was a high correlation between the relative intensity of the peak of the G4 of mutant aptamer (I_1656_/I_1099_), and the amount of GTX1/4 was similar to the linear relationship between the wild-type GO18 and GTX1/4 (Figure 5A). Therefore, the results convincingly demonstrated that the change in the SERS spectra of the aptamer was caused by the conformational change of the aptamer induced by GTX1/4. Furthermore, by comparing the change in the intensity of bands, we found that the order was 12C > 7A12A > GO18, and aptamers 12C and 7A12A have almost the same behavior to the GTX1/4. However, this good relationship was not found in the mutant aptamer 7G, 7A12T, and 12T (Figure 5B), which meant that the addition of GTX1/4 had no obvious effect on the conformation of other mutant aptamers.

To further verify the reliability of the results, the MST was used to determine the binding free energy of the mutant aptamer and GTX1/4. The results are shown in Figure 5C. The K_d_ values of 12C, 7A12A, 7G, 7A12T, and 12T were calculated to be 23.45 ± 13.00 nM, 23.72 ± 5.85 nM, 143.95 ± 32.94 nM, 151.96 ± 20.08 nM, and 223.56 ± 28.51 nM, respectively. Therefore, the order of affinity was 7A12A > 12C > 7G > 7A12T > 12T. The binding energy change of 100 ns under the binding state of the mutated aptamer and GTX1/4 ligand were calculated using MD (Figure 5D). The lower the binding free energy, the stronger the binding strength and the better the binding affinity between aptamers and target molecules. The above results have shown that the results of SERS, MST, and MD have been mutually verified and have provided further evidence for the results of the SERS analysis, indicating that the bases 12’ and 7’ played an important role in the binding of GTX1/4 to the aptamer.

#### 2.3.2. Optimization of Truncation

In consideration of the fact that GTX1/4 binds to the G4 plane of the wild-type aptamer, and dT7 plays an important role in the binding of the aptamer to GTX1/4, we optimized the aptamer by removing the first five nucleotides at the 5’-end and named this truncated variant of GO18 as GO18T.

**Figure 6:**
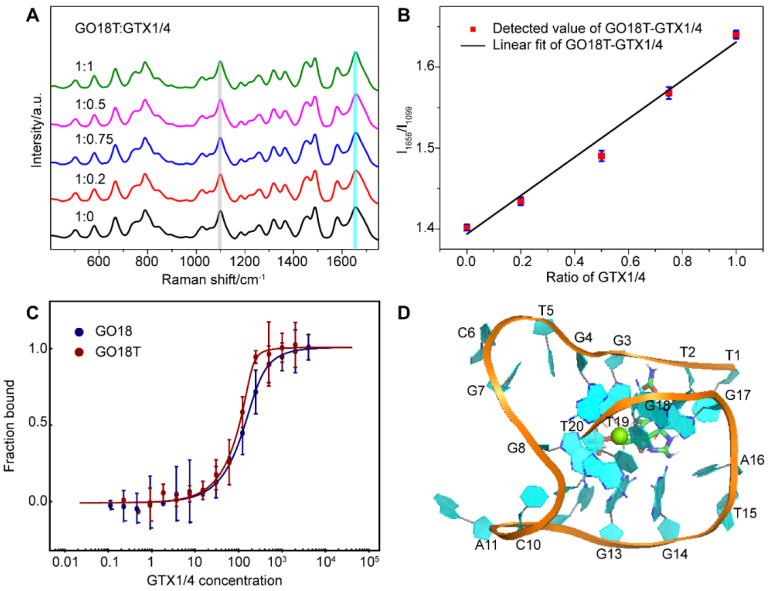
(**A**) SERS spectra of GO18T with different GTX1/4 ratios. All spectra were normalized to the PO_2_^−^ peak at 1099 cm^−1^ (grey bar). (**B**) The graph of relative intensity ratio (I_1656_/I_1099_) versus the addition ratio of GTX1/4. The error bar shows the standard deviation. (**C**) MST experiments of GO18T and GO18. (**D**) The complex structure of GO18T-GTX1/4 obtained by MD simulation.

Next, we added different proportions of GTX1/4 to the solution of GO18T to obtain its SERS spectra using the same method for GO18 (Figure 6A). The results (Figure 6B) showed that the relative intensity ratio (I_1656_/I_1099_) of the GO18T-GTX1/4 complex had a linear relationship with the amount of GTX1/4, which was similar to that of the wild-type GO18-GTX1/4 complex. This also indicated that the SERS method could also be used to evaluate the interaction between GO18T and GTX1/4. Furthermore, by comparing the extent of the SERS band intensity change, we found that the order was GO18T > GO18.

To further verify the SERS results, we used MST to measure the binding affinity of GO18T and GTX1/4, and calculated the K_d_ value as 5.29 ± 3.63 nM (Figure 6C). Thus, compared with that of GO18, the affinity of GO18t to GTX1/4 was increased by ~8 folds. Our K_d_ values of GO18 and GO18T are different from those of previous studies [26,41], which may be due to different buffer and detection methods.

MD simulation also further confirmed that the removal of the first five nucleotides enabled GO18T to combine with GTX1/4 more quickly and reach a stable state (Figure 6D). Exposure to the binding pocket of GO18 enhanced the spontaneous binding of GTX1/4 and GO18T, thus significantly improving the affinity of the two. It was, therefore, an effective optimization strategy to expose the binding sites (pocket) by removing some flexible fragments near the pocket.

Similar to the results of the part mutation optimization, the results of SERS, MST, and MD were also consistent for the truncation optimization, which strongly supported the accuracy and reliability of SERS results. Herein, we only studied the truncation and mutation of the two most commonly used aptamer optimization methods, which can also be used in other optimization methods in theory, and related research is underway. 

Compared with the experimental MST method, the SERS method established in this paper cannot directly provide quantitative measurement of binding affinity, but this study is an innovative application of the SERS method in G-quadruplex aptamer optimization. It can not only obtain the characteristic structure information and key binding site information of aptamer and ligand, which is helpful for the structural optimization of aptamers, but can also analyze the relative strength of the interaction between aptamer and GTX1/4, so as to obtain the optimized aptamer with high affinity, thus accelerating the aptamer optimization process. 

In addition, due to the high sensitivity of SERS technology to G4, and the simple experiment and data processing, this method can economically realize the rapid primary screening of aptamers, which is very helpful for difficult to obtain and expensive marine toxins. Moreover, these optimized aptamers may be further developed into biosensors for GTX1/4 diagnosis or inhibitors.

## 3. Materials and Methods

### 3.1. Reagents

All DNA aptamers were purchased from Sangon Biotech Co., Ltd. (Shanghai, China). The aptamer sequences are listed in Table S1. GTX1/4 was purchased from the National Research Council, Canada (the concentration was 75 μM), and GTX1 and GTX4 were 57 μM and 18 μm. Buffer solution (20 mM Tris-HCl, 100 mM NaCl, 5 mM KCl, 2 mM MgCl_2_, pH 7.5) was supplied by Tiandz (Beijing, China). KI, MgSO_4_, D_2_O, and Tween 20 were purchased from Sinopharm Chemical Reagent Co., Ltd (Shanghai, China). All solutions were prepared using Milli-Q ultrapure water. All chemical reagents were of analytical grade and were used without further purification.

### 3.2. Preparation of Ag IMNPs

According to Lee’s method [68], 10 mL of the silver sol was centrifuged (7000 rpm 10 min), and the supernatant was removed. Exactly 15 μL of the centrifuged silver sol was added to 15 μL of 1 mM KI solution. After incubation at 25 °C for 30 min, 3 mL of aptamer solution or aptamer-GTX1/4 mixed solution and 2 mL of 0.01 M MgSO_4_ were successively added to prepare the sample for Raman detection. The concentration of the aptamer in the final solution was 8.57 μM.

### 3.3. SERS Detection

Aptamer samples and GTX1/4 were prepared in a buffer (20 mM Tris-HCl, 100 mM NaCl, 5 mM KCl, 2 mM MgCl_2_, pH 7.5) unless otherwise stated. DNA and GTX1/4 solutions were added into the mixed solution at concentration ratios of 1:0, 1:0.2, 1:0.5, 1:0.75, and 1:1, and the concentration of DNA in all solutions was 50 μM. The samples were heated at 95 °C for 10 min, then quenched in an ice bath for 5 min, and finally allowed to stand at room temperature for 5 min for SERS detection.

A 10 μL sample was placed into a quartz capillary (0.7 cm inner diameter, 1.0 cm outer diameter). After focusing the laser on the liquid surface of the capillary, SERS spectra were collected using a K-sens Raman system (gora confocal Raman spectroscopy, ideaoptics, China). The laser wavelength was 532 nm, the integration time was 10 s, and the collection number was five times. The data preprocessing methods included spectral Savitzky–Golay polynomial fitting (window size was nine points), and baseline correction. The 2DCOS calculation [69,70] was completed via self-coding using MATLAB 7.0 software (MathWorks, Natick, MA, USA) and drawing using Origin 9.0 software.

### 3.4. MD Simulation

According to Song’s method [41], all MD simulations were carried out using the Gromacs−5.1.4 software. The simulation was based on the AMBER99bsc1 force fields and simple point charge water model. In each simulation, the aptamer model was placed in the center of the water box, and the distance from its surface to the boundary was more than 15 Å. The GTX1/4 molecule was prepared via the general AMBER force field using the antechamber, and its topology file and coordinate file were generated using the LEaP program in AmberTools 17 and transformed from ACPYPE to a GROMACS compatible file. In the simulation, Mg^2+^ and Cl^−^ were added to mimic a neutral environment, and the salt concentration was 150 mM. Periodic boundary conditions were used in the simulation. The particle mesh Ewald method was used to calculate the electrostatic interaction. The cut-off distance between the electrostatic force and van der Waals force was 14 Å. The LinCS algorithm was used to constrain all the keys in the system, and the time step was 2.0 fs. The pressure was maintained at 1 atm using the Berendsen method and the temperature was maintained at 25 °C using the velocity rescaling method. The duration of each simulation was 500 ns. The number of hydrogen bonds between groups in the G-tetrad was calculated using the gmx-hbond in GROMACS. The binding free energy of GTX1/4 with each aptamer was calculated by Autodock semiempirical free energy field and the Python program in AutodockTools.

### 3.5. Circular Dichroism

Aptamer samples at concentrations of 25 μM were treated the same way described in Section 3.3 and detected at 25 °C. CD spectra were recorded using a Chirascan spectrometer (Applied Photophysics, Ltd., Surrey, UK) with 0.1 cm path length quartz cuvettes at 25 °C. Scanning from 200 to 360 nm was performed at a scan speed of 120 nm/min. Each spectrum was an average of three scans. Data were buffer-subtracted and normalized to provide molar ellipticity values. In the magnesium ion contrast experiment, the aptamer solution was mixed with 10 mM MgSO_4_ solution for 30 min, and then CD was detected.

### 3.6. Determination of Affinity of MST 

To detect the binding affinity between GTX1/4 and aptamers, MST experiments [41] were performed on a Monolith NT.115 system (NanoTemper Technologies, Germany) using 10% LED- and 40% IR-laser power. The on and off time of the laser was set at 30 and 5 s, respectively. A two-fold dilution series for unlabeled ligand GTX1/4 was prepared. Equal volumes of 6-FAM-labeled aptamers at the 3’-end (400 nM) were added, resulting in GTX1/4 concentrations ranging from 0.12 to 4000 nM with a constant aptamer concentration of 200 nM in the binding buffer (20 mM Tris-HCl, 100 mM NaCl, 5 mM KCl, 2 mM MgCl_2_, pH 7.5, supplemented with 0.05% Tween 20). Aptamers were treated in the same way as described in Section 3.3 and detected at 25 °C. Experiments were performed at least three times, and the data were imported into the MO. Affinity Analysis Software to calculate the K_d_ value of the ligand binding to the aptamer.

## 4. Conclusions

The SERS technique established in this paper can provide a good spectrum and the characteristic band of G4 of the aptamer. Through the analysis of the one-dimensional SERS spectrum and 2DCOS of aptamer-GTX1/4 complex, we revealed that GTX1/4 acts on the G4 plane of GO18, and the corresponding structural change can also be observed. When analyzing the interaction between the optimized aptamer and GTX1/4, we found that the order of the intensity change for the aptamer G4 characteristic band was consistent with the order of the affinity calculated using MST and MD. Therefore, the SERS method can be used to reveal the information of key binding sites to guide the post-SELEX optimization of aptamers and evaluate the relative affinity between aptamers and ligands to obtain high affinity aptamers. Although this study only used truncated and mutated aptamers as the most commonly used optimization methods, it also serves as a reference for other optimization methods. Meanwhile, the selectivity of this method needs further investigation using other marine toxins. However, the SERS method established in this study is unlabeled and does not require the immobilization of small molecules or aptamers, which provides a new, rapid, highly sensitive, and economical platform for post-SELEX optimization of aptamers. At the same time, because it is difficult to obtain marine toxins, the selectivity of this method needs further investigation. However, it is also an important reference for biological research of other nucleic acid sequences containing G4 structures besides aptamers. Moreover, the methodologies presented in this paper have the potential to monitor the SELEX screening process because of its quantitative and structural characterization functions.

## Data Availability

Not applicable.

## Supplementary Materials

The following supporting information can be downloaded at: https://www.mdpi.com/article/10.3390/toxins14090622/s1, Table S1. Names and sequence of single-stranded DNA. Table S2. The bands and their assignment appeared in the SERS spectra of G-quadruplex GO18.
