# Supplementary material for: Optimization of Gonyautoxin1/4-Binding G-Quadruplex Aptamers by Label-Free Surface-Enhanced Raman Spectroscopy
Source: Toxins (Basel). 2022 Sep 6;14(9):622. doi: 10.3390/toxins14090622 (PMC9505997; doi:10.3390/toxins14090622)
Supplement: Supplementary file 1 [file toxins-14-00622-s001.zip › toxins-1846806-supplementary.pdf]

Supporting information for

Optimization of Gonyautoxin1/4-binding G-quadruplex Aptamers by Label-free Surface-enhanced Raman Spectroscopy

Yan Liu [1,2,†], Chengshun Jiang [2,3,†], Menghua Song [4,5], Yongbing Cao [6], Qiang Huang [4,5,*] and Feng Lu [1,2,*]

1 Department of Pharmaceutical Analysis, School of Pharmacy, Naval Medical University, 200433 Shanghai, China

2 Shanghai Key Laboratory for Pharmaceutical Metabolite Research, Naval Medical University, Shanghai 200433, China

3 Department of Pharmaceutical Analysis, School of Pharmacy, Fujian University of Traditional Chinese Medicine, Fuzhou 350108, China

4 State Key Laboratory of Genetic Engineering, Shanghai Engineering Research Center of Industrial Microorganisms, MOE Engineering Research Center of Gene Technology, School of Life Sciences, Fudan University, Shanghai 200438, China

5 Multiscale Research Institute of Complex Systems, Fudan University, Shanghai 201203, China

6 Institute of Vascular Disease, Shanghai TCM-Integrated Hospital, Shanghai University of Traditional Chinese Medicine, 200082 Shanghai, China

* Correspondence: huangqiang@fudan.edu.cn (Q.H.); fenglu@smmu.edu.cn (F.L.)

† These authors have contributed equally to the work.

Table S1. Names and sequence of single-stranded DNA

Name	Sequence（5′-3′）
GO18	AACCTTTGGTCGGGCAAGGTAGGTT
12C	AACCTTTGGTCCGGCAAGGTAGGTT
7A12A	AACCTTAGGTCAGGCAAGGTAGGTT
7A12T	AACCTTAGGTCTGGCAAGGTAGGTT
12T	AACCTTAGGTCTGGCAAGGTAGGTT
7G	AACCTTGGGTCGGGCAAGGTAGGTT
GO18T	TTGGTCGGGCAAGGTAGGTT

Table S2. The bands and their assignment appeared in the SERS spectra of G-quadruplex GO18

Raman Shift/cm^{-1}	Assignment
500	ν_s PO_2^-, dT, dG
581	dG
668	dG C2′-endo/*syn*
789	ν_s OPO, dT ring br
1022	d (νC-O)
1099	ν_s PO_2^-, bk
1259	dT, dGδNH(N2)
1317	dG C2′-endo/*syn*
1364	dG C2′-endo/*anti*
1487	dG N7 strong Hoogsteen H-bond
1581	dG N2H interbase H-bond
1656	dG O6 interbase H-bond

Abbreviations: d, deoxyribose; ν, stretch; δ, deformation; br, breathing; s, symmetric; bk, backbone.
